# Dopamine Signaling Leads to Loss of Polycomb Repression and Aberrant Gene Activation in Experimental Parkinsonism

**DOI:** 10.1371/journal.pgen.1004574

**Published:** 2014-09-25

**Authors:** Erik Södersten, Michael Feyder, Mads Lerdrup, Ana-Luisa Gomes, Hanna Kryh, Giada Spigolon, Jocelyne Caboche, Gilberto Fisone, Klaus Hansen

**Affiliations:** 1 Biotech Research and Innovation Centre (BRIC) and Centre for Epigenetics, University of Copenhagen, Copenhagen, Denmark; 2 Department of Neuroscience, Karolinska Institutet, Stockholm, Sweden; 3 INSERM, U952, CNRS UMR 7224, Université Pierre et Marie Curie, Paris 06, Paris, France; Friedrich Miescher Institute, Switzerland

## Abstract

Polycomb group (PcG) proteins bind to and repress genes in embryonic stem cells through lineage commitment to the terminal differentiated state. PcG repressed genes are commonly characterized by the presence of the epigenetic histone mark H3K27me3, catalyzed by the Polycomb repressive complex 2. Here, we present *in vivo* evidence for a previously unrecognized plasticity of PcG-repressed genes in terminally differentiated brain neurons of parkisonian mice. We show that acute administration of the dopamine precursor, L-DOPA, induces a remarkable increase in H3K27me3S28 phosphorylation. The induction of the H3K27me3S28p histone mark specifically occurs in medium spiny neurons expressing dopamine D1 receptors and is dependent on Msk1 kinase activity and DARPP-32-mediated inhibition of protein phosphatase-1. Chromatin immunoprecipitation (ChIP) experiments showed that increased H3K27me3S28p was accompanied by reduced PcG binding to regulatory regions of genes. An analysis of the genome wide distribution of L-DOPA-induced H3K27me3S28 phosphorylation by ChIP sequencing (ChIP-seq) in combination with expression analysis by RNA-sequencing (RNA-seq) showed that the induction of H3K27me3S28p correlated with increased expression of a subset of PcG repressed genes. We found that induction of H3K27me3S28p persisted during chronic L-DOPA administration to parkisonian mice and correlated with aberrant gene expression. We propose that dopaminergic transmission can activate PcG repressed genes in the adult brain and thereby contribute to long-term maladaptive responses including the motor complications, or dyskinesia, caused by prolonged administration of L-DOPA in Parkinson's disease.

## Author Summary

In Parkinson's disease (PD) the motor impairment produced by the progressive death of midbrain dopaminergic neurons is commonly treated with the dopamine precursor, L-DOPA. Utilizing a mouse model of PD, we show that L-DOPA, via activation of dopamine D1 receptors, promotes the expression of genes normally repressed by Polycomb group (PcG) proteins. We propose that this effect is exerted by promoting the phosphorylation of histone H3 on serine 28 at genomic regions marked by tri-methylation of the adjacent lysine 27, generating a H3K27me3S28p double-mark. This event leads to displacement of PcG proteins and aberrant gene expression. These findings reveal a previously unrecognized plasticity of PcG-repressed genes in terminally differentiated neurons. Furthermore, the identification of specific genes whose expression is increased upon prolonged treatment with L-DOPA and the consequential activation of dopamine D1 receptors offer a possibility to design novel therapeutic strategies to treat Parkinson's disease and potentially other disorders caused by dysfunctional dopaminergic transmission in the brain, such as drug addiction and schizophrenia.

## Introduction

An emerging concept in neurobiology is that many mechanisms implicated in chromatin remodeling and developmental processes retain their plasticity in the adult brain. Indeed, a number of environmental stimuli are known to generate chromatin modifications that have been causally linked to synaptic plasticity and associated behavioral and pathological responses. In this context, core histone modifications [1] have been implicated in cognitive functions, as well as in psychiatric conditions [1], [2].

Polycomb group (PcG) proteins maintain cell type specific gene repression that is established during early embryonic development by regulating chromatin structure [3]. The Polycomb repressive complex 1 (PRC1) mediates histone H2A lysine 119 mono-ubiquitination (H2AK119ub), while PRC2 di- and tri-methylates histone H3 lysine 27 (H3K27me2/3) [4], [5]. Functionally, both PRC1 and PRC2 can be recruited to genomic regions through direct binding to H3K27me3 marked chromatin. Importantly, while dysregulation of PcG binding to target genes has been implicated in serious developmental defects and diseases such as cancer [6], [7], aberrant derepression of PcG target genes have not been associated with pathology of terminally differentiated neurons [2].

Parkinson's disease (PD) is caused by the death of midbrain neurons producing dopamine. This disorder is commonly treated with L-DOPA, which upon conversion to dopamine, relieves the motor symptoms of PD [8]. However, prolonged use of L-DOPA results in the emergence of dyskinesia, involving dystonic and choreic movements [9]. Several lines of evidence indicate that L-DOPA-induced dyskinesia (LID) is caused by abnormal activation of dopamine D1 receptors (D1Rs) located on the medium spiny neurons (MSNs) of the striatum [10], [11]. This leads to increased gene expression through sequential activation of PKA, dopamine- and cAMP-regulated phosphoprotein of 32 kDa (DARPP-32), extracellular signal-regulated kinases (Erk), mitogen- and stress-activated kinase 1 (Msk1) and eventually phosphorylation of histone H3 at serine 10 (H3S10p) [12]–[15].

While the regulation of histone H3S10 phosphorylation has been studied in the adult brain [16], [17], almost nothing is known regarding H3S28 phosphorylation in neurons. However, in non-proliferating human fibroblasts it has been shown that H3K27me3S28 phosphorylation in response to MSK activation can lead to transcription of otherwise stably repressed genes [18]. The initial derepression is caused by displacement of gene repressor complexes containing PcG proteins, followed by transcriptional activation.

In this study, we describe an important link between dopamine signaling, H3K27me3S28 phosphorylation, and aberrant gene expression associated to reduced PcG binding. Using a mouse model of PD, we show that dopamine via D1Rs increases H3K27me3S28 phosphorylation in striatal MSNs via two pathways: 1) activation of Msk1, leading to phosphorylation of H3K27me3S28 and 2) activation of DARPP-32 leading to protein phosphatase 1 (PP1) inhibition and suppression of H3K27me3S28p dephosphorylation. The combined effect is an accumulation of H3K27me3S28p at gene promoters that reduces PcG binding and allows transcription of a subset of genes. The results reveal a previously unrecognized plasticity of PcG-repressed genes in the adult brain, which upon environmental changes can be aberrantly induced via Erk-Msk1 mediated H3K27me3S28 phosphorylation and PKA-DARPP-32-dependent modulation of PP1 activity towards the same histone mark.

## Results

### L-DOPA triggers H3K27me3S28 phosphorylation in MSNs of hemiparkinsonian mice

**Figure 1:**
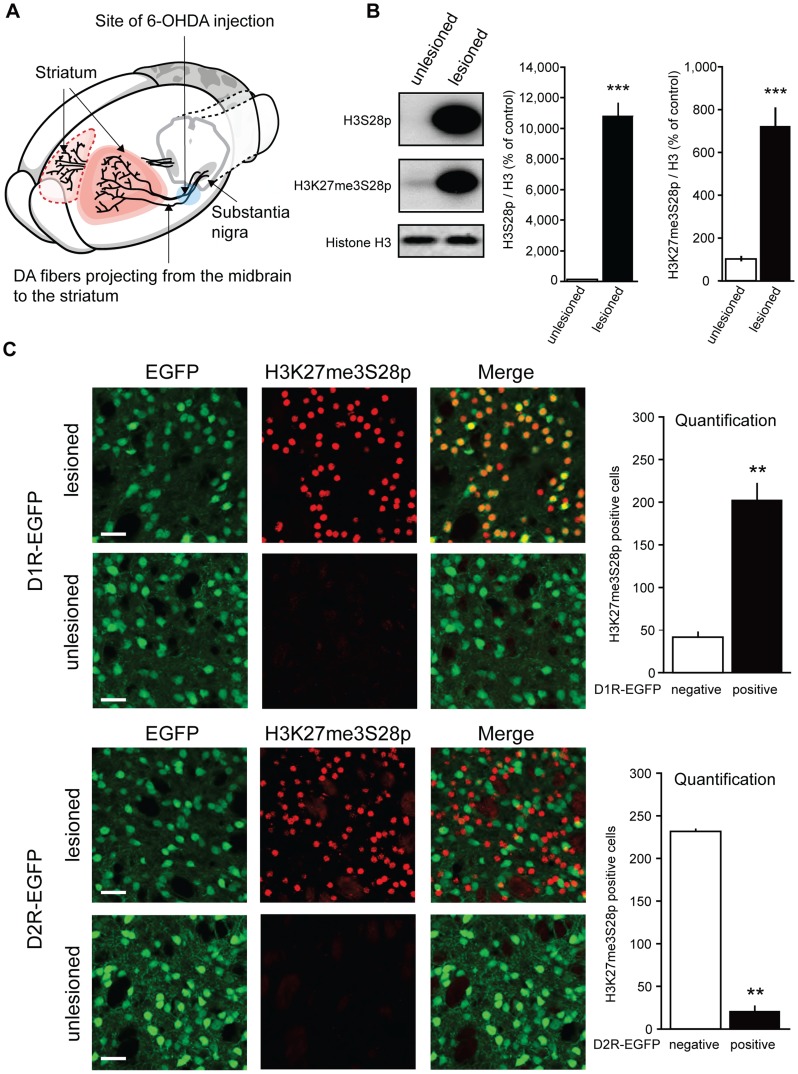
L-DOPA triggers H3K27me3S28 phosphorylation in MSNs of hemiparkinsonian mice. A) Hemiparkinsonian mice were generated by unilateral injection of 6-OHDA into the medial forebrain bundle, eliminating the dopaminergic (DA)-innervation from the substantia nigra into the striatum. B) Acute L-DOPA administration (1 hour) increased H3S28p and H3K27me3S28p in the lesioned striatum shown by Western blotting. Mean values ±S.E.M in the bar diagrams, *** p<0.001, n = 3–6 per condition. C) Confocal micrographs of H3K27me3S28p immunostaining from the striatum of D1- or D2-receptor EGFP-expressing mice. Acute L-DOPA administration increased H3K27me3S28p which predominantly co-localized with EGFP-labeled cells of D1R-EGFP mice (t_(4)_ = 8.20, ** p<0.01) and which segregated from EGFP-labeled cell bodies of D2R-EGFP mice (t_(2)_ = 30.30, ** p<0.01). n = 2–3 per condition. Quantifications are means ±S.E.M. Scale bar is 30 µm.

The ability of L-DOPA to activate the Erk-Msk1 pathway in striatal MSNs [15], [19], [20], led us to hypothesize that signaling through dopamine receptors would induce phosphorylation of S28 in the context of H3K27me3 marked genomic sites to generate the H3K27me3S28p double histone modification. To test this possibility we turned to an experimental mouse model of PD in which unilateral stereotaxic injection of the neurotoxin 6-OHDA results in the elimination of the dopaminergic innervation to the basal ganglia (Figure 1A) [20], [21]. In the lesioned striatum, MSNs react to the loss of dopamine by developing a remarkable sensitization to D1R agonists and, upon L-DOPA treatment strongly activate Msk1, while the MSNs in the contralateral, unlesioned striatum are unaffected [22]. This unilateral model of PD has the advantage that each mouse can serve as its own within-subject control as the dopamine sensitized MSNs in the striatum of the 6-OHDA lesioned side respond with intense D1R-mediated signaling to administration of L-DOPA, while the MSNs in the unlesioned side are not affected [15], [19], [20].

In the first experiment, lesioned mice were injected with L-DOPA and sacrificed 1 hour later (acute L-DOPA). By Western blotting, we observed that L-DOPA caused a dramatic increase in H3K27me3S28 phosphorylation in the lesioned striatum compared to the unlesioned striatum (Figure 1B).

The striatum contains two main populations of MSNs that are enriched for either D1Rs or dopamine D2 receptors (D2Rs). Activation of these neurons produces opposite behavioral responses, which are related to their distinct connectivity to the output stations of the basal ganglia [23]–[26]. To identify the population(s) of striatal MSNs that responded to L-DOPA with increased H3K27me3S28 phosphorylation we made use of transgenic mice expressing EGFP under the control of regulatory elements of the D1R or D2R (D1R-EGFP or D2R-EGFP) [27]. These mice were lesioned, treated with L-DOPA and perfused after 1 hour. The results showed that, in the lesioned striata of D1R-EGFP mice, H3K27me3S28p co-localized with the EGFP-labeled cell bodies (Figure 1C), while in D2R-EGFP mice, H3K27me3S28p was segregated from EGFP-labeled cell bodies (Figure 1C).

To further confirm that D1Rs activate the signaling cascade inducing the H3K27me3S28p mark, we injected naïve mice with the specific D1R-agonist SKF81297. Indeed, H3K27me3S28p was increased 1 hour after SKF81297 injection (Figure S1A).

We concluded that in hemiparkisonian mice, where the dopamine innervation was eliminated by unilateral injection of 6-OHDA, acute administration of L-DOPA produced a large increase in H3K27me3S28p specifically localized to the nuclei of striatal D1R-MSNs.

### L-DOPA mediated H3K27me3S28 phosphorylation correlates with reduced PcG protein binding and activation of genes

**Figure 2:**
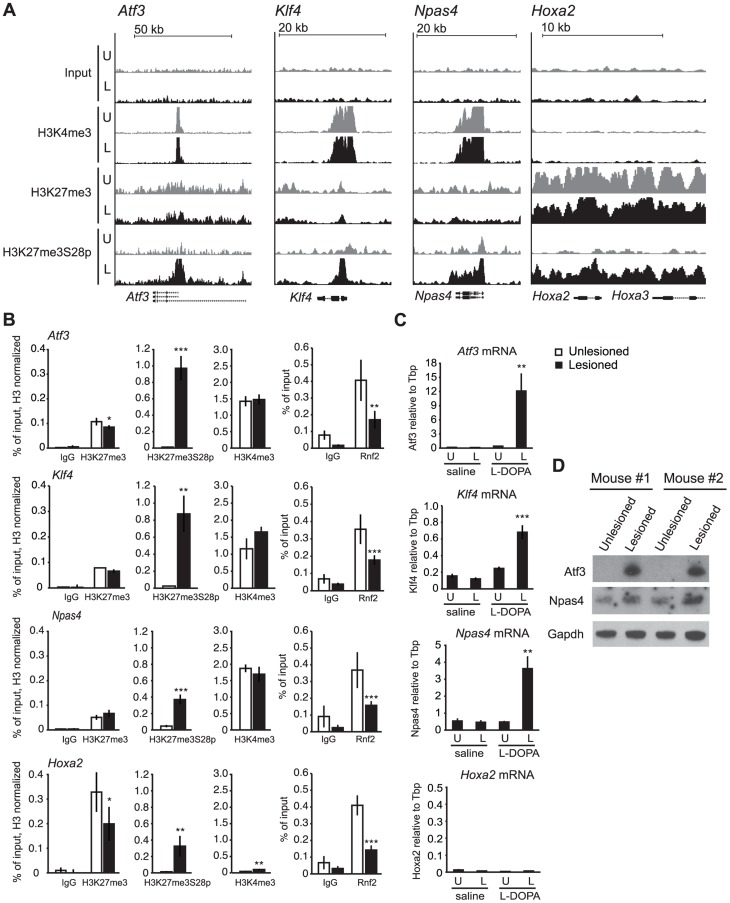
L-DOPA mediated H3K27me3S28 phosphorylation correlate with reduced PcG binding and derepression of genes. A) ChIP-seq signals for input chromatin, H3K4me3, H3K27me3 and H3K27me3S28p in unlesioned and lesioned striata after acute L-DOPA. Shown are genomic regions at the *Atf3*, *Klf4*, *Npas4* and *Hoxa2* genes. B) ChIP-qPCR on chromatin from unlesioned and lesioned striata after acute L-DOPA. Signals from histone marks were normalized to the total histone H3 using the same primers. Error bars represent standard deviation calculated from triplicate qPCR reactions. The signal in the lesioned striatum was compared to the signal in the unlesioned striatum, * p<0.1, ** p<0.05, *** p<0.001. C) RT-qPCR using primers for *Atf3*, *Klf4*, *Npas4*, *Hoxa2* and *Tbp* (used for normalization) showing mRNA levels of the genes in saline control or acute L-DOPA (n = 3), in unlesioned and lesioned striata, respectively. Data are means ±S.E.M of the biological triplicates. *p<0.05, **p<0.01, ***p<0.001. D) Western blot showing induced protein levels for Atf3 and Npas4 in lesioned compared to unlesioned striata after acute L-DOPA treatment of two mice. Gapdh was used as loading control.

Given the dramatic increase in H3K27me3S28p in D1R expressing MSNs, we examined the global distribution of this mark on chromatin. We undertook chromatin immunoprecipitations (ChIPs) for H3K27me3S28p from pooled lesioned or unlesioned striata, after acute administration of L-DOPA (tissue from 45 mice were pooled for each condition), followed by ChIP-sequencing (ChIP-seq). This was also done using antibodies for H3K4me3 and H3K27me3, to define potentially active or PcG-repressed chromatin, respectively. In this way, we identified four genes encoding transcription factors (TFs) where H3K27me3S28 phosphorylation was induced near the transcription start sites (TSS) (Figure 2A). Two of them, *Atf3* and *Npas4* have previously been found to be implicated in neuronal plasticity, while *Klf4* and *Hoxa2* are well known PcG target genes implicated in stem cell function and cellular differentiation. In the lesioned striata the peaks observed for H3K27me3S28p at the *Atf3*, *Klf4* and *Npas4* genes coincided with the H3K27me3 peaks. Co-occurrence was also observed for H3K27me3S28p and H3K4me3 (Figure 2A). In contrast, chromatin at the *Hoxa2* locus was blanketed with H3K27me3 and H3K27me3S28p in the lesioned striata, without any apparent H3K4me3 signal (Figure 2A).

To support these observations, we performed ChIP-qPCR on chromatin from lesioned and unlesioned striata after acute L-DOPA. Antibodies against H3K27me3S28p, H3K27me3, H3K4me3 and Rnf2 (PRC1 subunit), and general IgG as a negative control were used (Figure 2B). The results confirmed the induction of H3K27me3S28p in the lesioned striatum upon L-DOPA stimulation and the presence of H3K27me3 at all genes analyzed, as well as H3K4me3 enrichment in chromatin from the unlesioned and lesioned striata on *Atf3*, *Klf4* and *Npas4* genomic loci. In line with our previous findings [18], the induction of H3K27me3S28 phosphorylation on these loci correlated with a reduction in Rnf2 binding.

Whereas L-DOPA induced H3K27me3S28 phosphorylation in the striatum was restricted to D1R-MSNs, the origins of signal in the H3K27me3 and H3K4me3 ChIPs were uncertain, due to the presence of several cell types in the tissue. We have estimated the contribution of D1R-MSNs to the bulk chromatin analyzed in our ChIPs to be approximately 43% (Figure S3). Therefore, D2R-MSNs could account for a significant part of the H3K4me3 and H3K27me3 signals detected by ChIP.

The increase in H3K27me3S28p and the reduced binding of Rnf2 suggested that these genes become de-repressed upon acute L-DOPA treatment. We therefore performed RT-qPCR using RNA isolated from lesioned mice treated with L-DOPA or saline as control (Figure 2C). Indeed, L-DOPA increased the expression of mRNA for *Atf3*, *Klf4* and *Npas4* in the lesioned compared to the unlesioned striatum and to the saline controls, whereas the expression of *Hoxa2* was unchanged (Figure 2C). The lack of expression from the *Hoxa2* locus was supported by the absence of the active H3K4me3 histone mark. Importantly, the increases of Atf3 and Npas4 were furthermore confirmed at the protein level (Figure 2D), suggesting the potential involvement of these TFs in the phenotypic effects produced by L-DOPA in parkinsonian mice.

In summary, our data suggest that administration of L-DOPA in a mouse model of PD promotes H3K27me3S28 phosphorylation on several PcG target genes marked by H3K27me3. This effect occurs in the striatal D1R-MSNs of the lesioned brain hemisphere and correlates with a reduction in Rnf2 binding and increased gene expression.

### Genome wide induction of H3K27me3S28 phosphorylation correlates with an increase of mRNA transcripts from H3K27me3 marked gene loci

**Figure 3:**
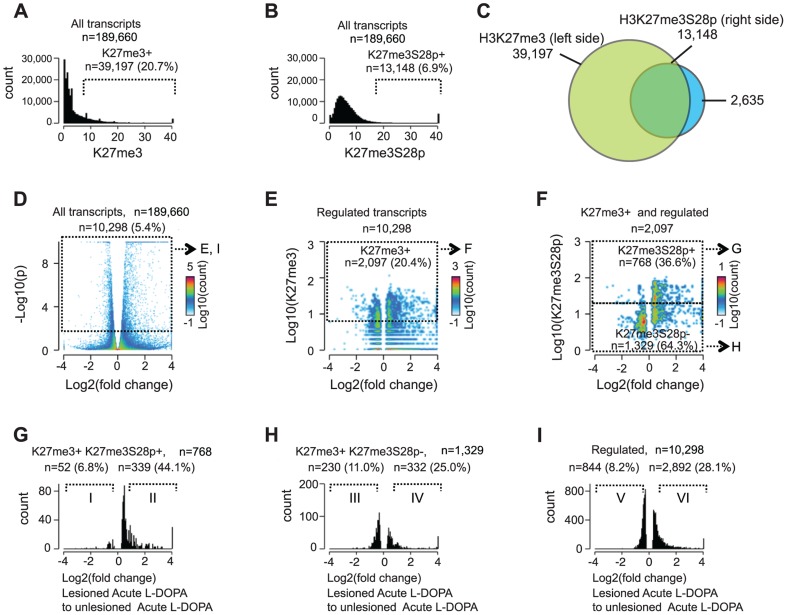
Induction of H3K27me3S28 phosphorylation genome wide correlates with an increase of mRNA transcripts from H3K27me3 marked gene loci. Genome-wide correlation of expression data obtained by RNA-seq and H3K27me3 and H3K27me3S28p ChIP-seq data from lesioned and unlesioned striata of acute L-DOPA treated mice. A) and B) Histogram of the levels of H3K27me3 in the unlesioned striata (A) or H3K27me3S28p in the lesioned striata (B) at the TSS of genes (+/−1,000 bp). Values on x-axis are H3K27me3 levels (A) or H3K27me3S28p levels (B) normalized to reads per 1 kbp per 10 million reads (see Experimental Procedures). Genes were scored H3K27me3 positive (A) or H3K27me3S28p positive (B), respectively. C) Venn diagram to show the overlap between gene loci in the mouse genome which can give rise to a mRNA transcript that are marked by H3K27me3 in the unlesioned tissue sample from the left brain hemisphere, that gain H3K27me3S28 phosphorylation in the lesioned right brain hemisphere upon acute L-DOPA administration. D) Volcano plot of differentially expressed transcripts in the lesioned striata compared to the unlesioned striata after acute L-DOPA. Marked areas indicate regulated transcripts. E) 2D-histogram of transcripts that were regulated. Values on the x-axis correspond to log2 fold change in the expression level of the transcript, whereas y-axis values correspond to the quantitated H3K27me3 levels at TSS of the corresponding gene (normalized to general H3). Of the regulated transcripts, 20.4% originated from genes that scored H3K27me3 positive according to the threshold mentioned in A (marked area). F) 2D-histogram of regulated transcripts originating from H3K27me3 positive genes. Values on the x-axis correspond to log2 fold change in the expression level of the transcript, y-axis levels correspond to the quantitated, normalized, H3K27me3S28p levels at TSS of the corresponding gene. G–I) Histograms of the fold change in expression of the H3K27me3S28p positive and H3K27me3 positive genomic loci (G), H3K27me3S28p negative, but H3K27me3 positive genomic loci (H), and all regulated transcripts (I). H3K27me3S28p positive transcripts (G) are much more frequently induced than H3K27me3S28p negative (H) or regulated transcripts in general (I). Roman numerals indicate the subpopulations used for Gene Ontology Enrichment Analysis (Figure S4).

To estimate the genome-wide extent of H3K27me3S28 phosphorylation induced by L-DOPA, we scored regions +/−1 kb from the transcription start sites (TSS) of all annotated (mm9) mouse transcripts (n = 189,660) for the enrichment of H3K27me3 in chromatin from unlesioned striata (Figure 3A) and H3K27me3S28p in lesioned striata (Figure 3B). For this correlation, the H3K27me3 mark was determined in the unlesioned striata, instead of the lesioned striata, in order to ensure that the H3K27me3 signal was not altered by epitope masking due to H3K27me3S28 phosphorylation [18]. Using a cutoff where only 5% of the regions were expected to score positive by chance (see Materials and Methods), this analysis showed that approximately 20.7% (n = 39,197) of all loci that can give rise to mRNA transcripts were H3K27me3 positive (Figure 3A) and 6.9% (n = 13,148) were H3K27me3S28p positive (Figure 3B). As observed in the Venn diagram presented in Figure 3C the majority (83%) of H3K27me3S28 phosphorylation occurred in genomic regions that were already marked by H3K27me3 before L-DOPA administration, while approximately 17% of the genomic sites had levels of H3K27me3 below our defined cut-off for the analysis and could in principle have gained H3K27me3 upon L-DOPA administration.

Having observed that an impressive 33.5% of all H3K27me3 positive loci that potentially could give rise to mRNA transcripts became enriched for H3K27me3S28p upon L-DOPA stimulation, we next asked if and to which extent these transcripts were actually induced. We therefore performed global RNA-sequencing (RNA-seq) on mRNA isolated lesioned mice treated with L-DOPA. The analysis showed that 1 hour after L-DOPA, 5.4% (n = 10,298) of all transcripts had significantly changed expression in the lesioned striatum compared to the unlesioned striatum (Figure 3D). Importantly, 20.4% of these transcripts also scored positive for H3K27me3 at the chromatin level (Figure 3E; threshold mentioned in Figure 3A). We next examined to which extent the regulated transcripts that were marked by H3K27me3 at their genomic loci also scored positive for H3K27me3S28p (according to Figure 3B) and found that 36.6% matched this criterion (Figure 3F). It was furthermore apparent that most transcripts with high H3K27me3S28p levels near the TSS were induced rather than repressed. Further analysis of the different populations of regulated transcripts showed that, of the 768 that originated from H3K27me3- and H3K27me3S28p-positive loci, 52 were ≥1.5 fold down-regulated, whereas the majority 339 were ≥1.5 fold up-regulated (Figure 3G). This could be compared to the 1,329 regulated transcripts originating from H3K27me3 positive loci lacking H3K27me3S28p of which 230 were ≥1.5 fold down-regulated and 332 were up-regulated (Figure 3H), or to all 10,298 regulated transcripts (regardless of specific histone marks at their loci), where 844 were ≥1.5 fold down-regulated and 2,892 were ≥1.5 fold up-regulated (Figure 3I).

Altogether, regulated transcripts originating from H3K27me3 positive loci that gained the H3K27me3S28p mark were more frequently induced (87%, Figure 3G) than regulated transcripts originating from H3K27me3 positive loci not gaining the H3K27me3S28p mark (59%, Figure 3H) or regulated transcripts in general (77%, Figure 3I). These data highlight a clear correlation between induction of H3K27me3S28 phosphorylation at specific genomic loci and increased transcription in response to acute L-DOPA stimulation in the lesioned striata of parkisonian mice.

**Figure 4:**
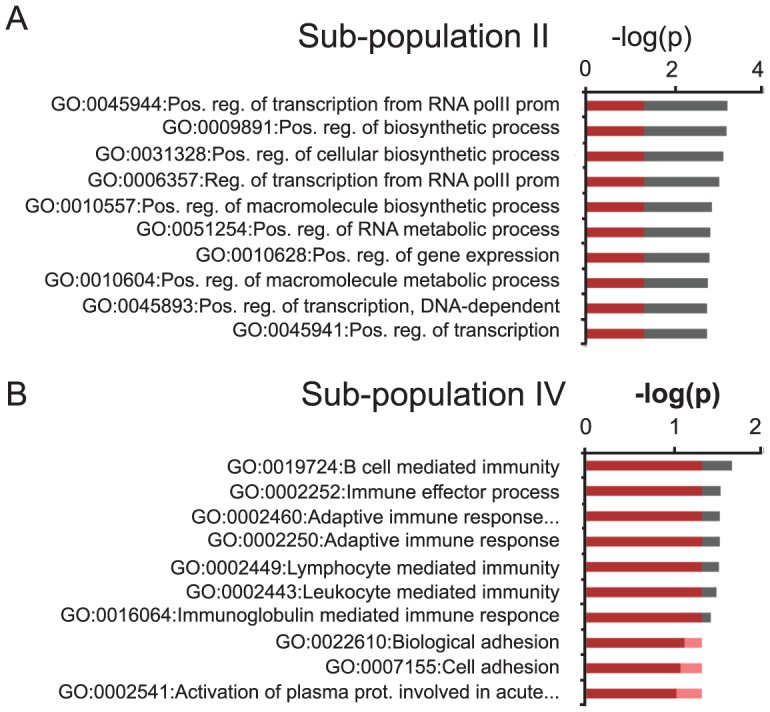
GO-analysis of specific subgroups. A–B) GO-terms of significantly up-regulated H3K27me3 and H3K27me3S28p positive transcripts in the sub-populations indicated with roman numerals II (A) and IV (B) in Figure 3G and H. Values on the axis are −log(p-values) of the most significant GO-terms (Benjamini-Hochberg corrected for multiple testing). Red coloring covers the values below 0.05 (approximately −1.3 when log10 transformed). For GO-terms of all subgroups I–VI, please see Figure S4.

Gene ontology (GO) analysis of the group of transcripts up-regulated 1.5 fold or more originating from H3K27me3 positive loci that gained the H3K27me3S28p mark (roman numerical II in Figure 3G), suggested that up-regulation of these gene products could affect overall transcriptional activity and rate of biosynthesis in neuronal cells (Figure 4A, Figure S4 and Table S1). This was in contrast to the group of up-regulated transcripts (≥1.5 fold) originating from H3K27me3 positive loci that did not gain H3K27me3S28 phosphorylation, which enriched for GO-terms involved in immune response (Figure 4B, Figure S4, subpopulation IV).

### H3K27me3S28 phosphorylation is controlled by Msk1 and DARPP-32-mediated inhibition of protein phosphatase 1 (PP1)

We have previously shown that MSK1 and MSK2 are the kinases mediating H3K27me3S28 phosphorylation in human fibroblasts, as pharmacological inhibition or shRNA mediated knockdown of MSK1 and MSK2 prevented the induction of H3K27me3S28p [18]. To elucidate which molecular pathways downstream of D1R activation in MSNs are implicated in the regulation of H3K27me3S28 phosphorylation, we lesioned Msk1−/− mice with 6-OHDA and injected them with L-DOPA. In line with our previous findings in fibroblasts, induction of H3K27me3S28p in the lesioned striatum was reduced in Msk1−/− mice compared to wt mice (Figure S5).

**Figure 5:**
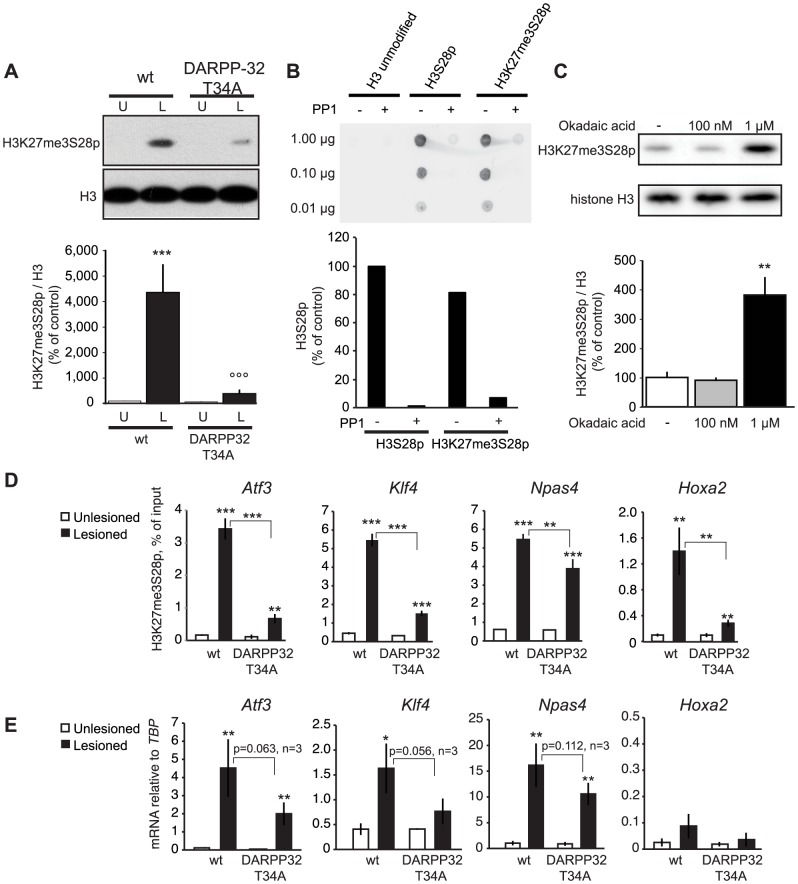
L-DOPA induced H3K27me3S28 phosphorylation and gene activation are dependent on a functional PP1 interaction site on DARPP-32. A) H3K27me3S28p was attenuated in T34A mutant mice following chronic administration of L-DOPA (lesioned x genotype interaction: F_(1,28)_ = 12.21, p = 0.002). *** p<0.001 vs unlesioned wt hemisphere, °°° p<0.001 vs lesioned wt hemisphere. N = 8 per condition. Data are means ±S.E.M.). B) PP1 efficiently dephosphorylates H3K27me3S28p *in vitro*. H3 non-modified, H3S28p or H3K27me3S28p modified peptides were incubated in the presence or absence of PP1. The peptide dot-blots were developed using an anti-H3S28p antibody. C) Striatal tissue slice culture experiment showing that H3K27me3S28p was induced after 50 min incubation with 1 µM, but not 100 nM, okadaic acid (effect of treatment: F_(2,8)_ = 19.64, p = 0.0008). ** p<0.01 vs vehicle and 100 nM okadaic acid (n = 3–4). Data are means ±S.E.M. D) Levels of H3K27me3S28p on regulatory regions of *Atf3*, *Klf4*, *Npas4* and *Hoxa2* were attenuated in striata from DARPP-32 T34A mutant mice compared to wt mice. ChIP-qPCR for H3K27me3S28p from unlesioned and lesioned striata from DARPP-32 T34A mutant and wt mice (acute L-DOPA). Data are means +/−S.E.M. of triplicate qPCR reactions. The signal from the lesioned striata is compared to the respective signal in the unlesioned striata, and the signal in the DARPP-32 T34A chromatin is compared to the respective signal in wt chromatin; * p<0.05, ** p<0.01, *** p<0.001. E) Attenuated activation of genes in DARPP-32 T34A mice after L-DOPA injection. RT-qPCR using primers for *Atf3*, *Klf4*, *Npas4*, *Hoxa2* and *Tbp* (for normalization) showing mRNA levels of respective gene in unlesioned and lesioned striata of acute L-DOPA injected DARPP-32 T34A mutant or wt mice. N = 3 mice per condition, unlesioned and lesioned striata respectively. Data are means ±S.E.M of biological triplicates. * p<0.05, ** p<0.01, *** p<0.001.

The exaggerated D1R transmission induced by L-DOPA in the MSNs of the dopamine-depleted striatum is characterized by elevated cAMP production and PKA activity [11], [13]. PKA further relays the signal via phosphorylation of DARPP-32 at T34 [15], [28]. This converts DARPP-32 into an inhibitor of protein phosphatase 1 (PP1), thereby suppressing dephosphorylation of downstream PP1 targets [29]. We have previously shown that a T34A mutation on DARPP-32 decreases L-DOPA-induced phosphorylation of Erk and histone H3S10 [14]. Therefore, we examined whether DARPP-32-mediated inhibition of PP1 was also involved in the regulation of H3K27me3S28p. When lesioned mice harbouring a T34A mutation in DARPP-32 were injected with L-DOPA we observed a significant less pronounced H3K27me3S28 phosphorylation in comparison to wt mice (Figure 5A). This finding suggested that PP1 is involved in the dephosphorylation of H3K27me3S28p. To test this possibility, we conducted an *in vitro* phosphatase assay, in which H3 peptides, either unmodified or modified with S28p or K27me3S28p, were incubated with PP1. Changes in the phosphorylation of the peptides after the reactions were detected by dot-blotting using an H3S28p antibody (Figure 5B). This assay showed that PP1 could efficiently dephosphorylate H3K27me3S28p.

To confirm that PP1 is the phosphatase acting on H3K27me3S28p in the striatum, we examined the effect of okadaic acid, which inhibits PP1 and PP2A [30], on H3K27me3S28p in a slice preparation from striatum by Western blotting (Figure 5C). Incubation of striatal slices with 1 µM okadaic acid, a concentration that inhibits both PP1 and PP2A, was sufficient to change the equilibrium towards increased levels of H3K27me3S28p compared to vehicle. In contrast, a concentration of 100 nM okadaic acid, which inhibits PP2A but is insufficient for PP1 inhibition [30], did not affect H3K27me3S28p levels. This supported PP1 as a phosphatase removing S28p from the H3K27me3S28p double-marked chromatin *in vivo*.

Next, we analyzed the outcome of the global reduction in H3K27me3S28p on regulatory regions of specific genes. Lesioned DARPP-32 T34A and wt mice were treated with L-DOPA and striatal tissue from the lesioned and unlesioned striatum was analysed by ChIP-qPCR. The induction of H3K27me3S28p at the *Atf3*, *Klf4*, *Npas4* and *Hoxa2* genes was significantly reduced in chromatin from the lesioned striatum of DARPP-32 T34A mice compared to wt mice (Figure 5D). Notably, this reduction correlated with decreased expression of *Atf3*, *Klf4* and *Npas4* mRNA in the lesioned striatum of L-DOPA injected DARPP-32 T34A mice compared to wt mice (Figure 5E).

Overall these data suggested that D1R stimulation induces transcription associated to H3K27me3S28 phosphorylation via two parallel pathways: 1) activation of Erk-Msk kinases and 2) concomitant PKA-mediated DARPP-32-phosphorylation, leading to inhibition of PP1 and suppression of H3K27me3S28p dephosphorylation.

### Increased transcription from Polycomb-repressed genes in L-DOPA induced LID

LID is a serious motor complication caused by prolonged administration of L-DOPA to patients affected by PD [9]. This condition has been linked to persistent hyper-activation of the cAMP/DARPP-32 signaling cascade, produced by L-DOPA acting on sensitized D1Rs [10], [11]. Lesioned mice display dyskinetic behaviour in response to 9 sequential daily L-DOPA injections (chronic L-DOPA) [14], [15]. The severity of LID after chronic L-DOPA administration has been shown to correlate to the level of H3S10 phosphorylation and the induction of specific genes, such as *Fosb* [12], [15], [19], [31].

**Figure 6:**
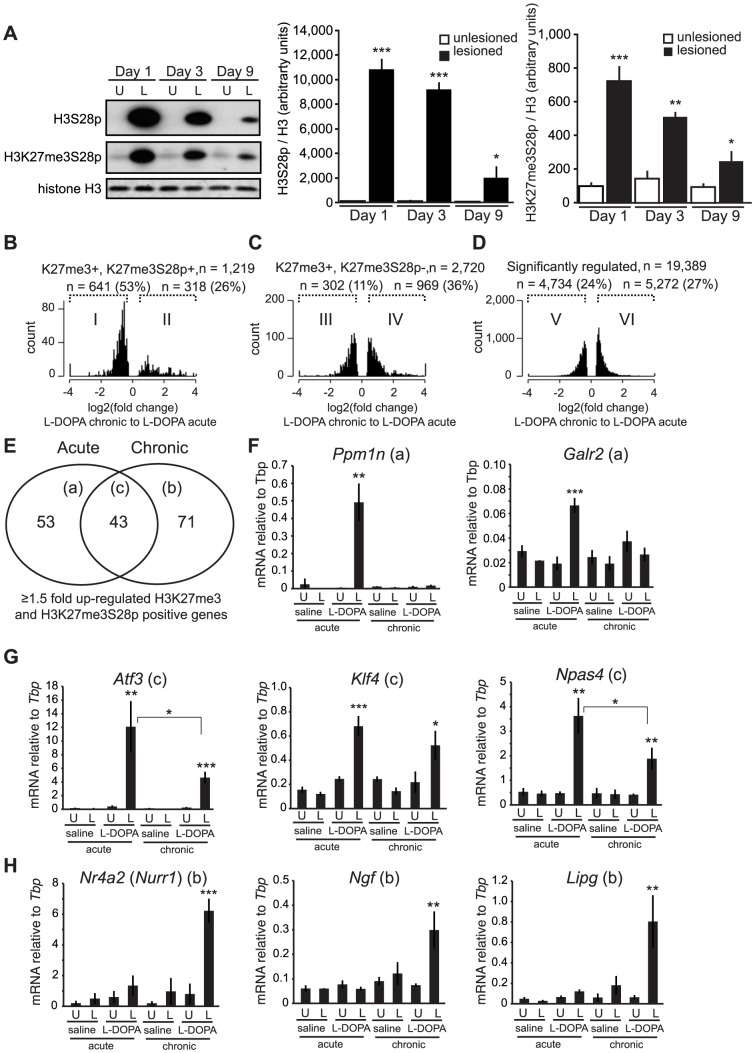
Chronic administration of L-DOPA decreases the induction of H3K27me3S28p and alters the transcriptomal response to L-DOPA. A) L-DOPA increased H3S28p and H3K27me3S8p in the lesioned striatum that progressively decreased following subsequent administrations (H3S28p: lesion x day interaction: F_(2,25)_ = 38.17, p<0.0001; H3K27me3S28p: lesion x day interaction: F_(2,24)_ = 11.42, p = 0.0003). *** p<0.001, ** p<0.01, * p<0.05 vs unlesioned hemisphere of the same day. n = 3–6 per condition. Data are means ±S.E.M. B–D) Histograms of differentially expressed transcripts in lesioned striata after chronic L-DOPA compared to acute L-DOPA, from H3K27me3S28p positive and H3K27me3 positive genes (B), from H3K27me3S28p negative but H3K27me3 positive genes (C), and from genes regardless of specific histone marks (D). E) Venn-diagram showing number of genes induced exclusively by acute (53; subgroup a) or chronic (71; subgroup b) and genes commonly induced after both acute and chronic L-DOPA administration (43; subgroup c). F–H) mRNA levels of (F) *Ppm1n*, *Galr2*, (G) *Atf3*, *Klf4*, *Npas4*, (H) *Nurr1*, *Ngf*, *Lipg* relative to *Tbp* in unlesioned and lesioned striata of acutely or chronically saline or L-DOPA injected mice determined by RT-qPCR. n = 3 mice per condition, unlesioned and lesioned striata respectively. Data are means ±S.E.M of the biological triplicates. *** p<0.001, ** p<0.01, * p<0.05. Small letters a–c following the gene names in brackets in panel F–H refers to the subgroups in the Venn diagram in panel E.

To investigate the contribution of H3K27me3S28 phosphorylation to the changes in gene expression associated to LID, 6-OHDA lesioned mice were treated chronically with L-DOPA and the levels of H3S28p and H3K27me3S28p were measured after 1, 3 and 9 days of administration. As expected, L-DOPA increased H3S28p and H3K27me3S28p in the lesioned striatum. However, the induction of these histone marks was progressively reduced during the course of chronic L-DOPA administrations (Figure 6A).

To examine if specific changes in gene expression associated to LID occurred for PcG-repressed genes, we performed global RNA-seq on mRNA isolated from striata of lesioned mice that had been treated chronically with L-DOPA (9 days). Transcripts that were significantly changed in the lesioned striatum after chronic L-DOPA were scored for enrichment of H3K27me3 in the unlesioned striatum and then for H3K27me3S28p in the lesioned striatum after acute L-DOPA, according to Figure 3A–F. From this analysis we concluded that despite the reduced induction of H3K27me3S28p following chronic administration of L-DOPA, regulated transcripts originating from genomic loci marked by H3K27me3 and H3K27me3S28p were still largely induced (76%, Figure S6A, subpopulation II) in comparison to regulated transcripts originating from genomic loci marked by H3K27me3 only (45%, Figure S6B, subpopulation IV) or in comparison to all regulated transcripts (46%, Figure S6C, subpopulation VI).

As the induction of the H3K27me3S28p mark was reduced after chronic L-DOPA compared to acute L-DOPA (Figure 6A), we next examined whether the transcripts from genes marked by H3K27me3 and H3K27me3S28p were less induced after chronic L-DOPA compared to acute L-DOPA treatment. Amongst the regulated transcripts originating from H3K27me3 and H3K27me3S28p positive genomic loci, 53% were ≥1.5 fold less expressed in chronic L-DOPA compared to acute L-DOPA (Figure 6B). In contrast, only 11% of regulated transcripts originating from H3K27me3 positive and H3K27me3S28p negative genomic loci (Figure 6C), and 24% of all regulated transcripts were less expressed in chronic L-DOPA compared to acute L-DOPA. These data suggested that the level of transcription from H3K27me3 and H3K27me3S28p positive genes correlated with the level of induced H3K27me3S28 phosphorylation.

Finally, we examined the individual genes that were ≥1.5 fold induced after chronic L-DOPA and scored positive for H3K27me3 and H3K27me3S28p. We found that a large number of these genes differed from those induced by acute L-DOPA (Figure 6E, Table S1 and Table S2). Thus, a total of 96 genes were induced after acute L-DOPA and 114 genes after chronic L-DOPA, but only 43 genes were commonly induced. To confirm this observation, we performed RT-qPCR on selected genes and could confirm, for instance, that *Ppm1n* and *Galr2* were induced after acute L-DOPA, but not after chronic L-DOPA or saline (Figure 6F; see also Figure S1B showing that the lesion alone does not induce H3K27me3S28p (vehicle control)). *Atf3*, *Klf4*, and *Npas4* were induced by both acute L-DOPA and, albeit to a lesser extent by chronic L-DOPA (Figure 6G), whereas *Nr4a2* (also known as *Nurr1*), *Ngf* and *Lipg* were only induced after chronic L-DOPA (Figure 6H). For these genes we could observe peaks in the ChIP-seq data for H3K27me3 in the lesioned and unlesioned striata, induced H3K27me3S28p in the lesioned striatum and H3K4me3 peaks in the lesioned and unlesioned striata (Figure S7).

As an example of a gene that was induced at the protein level in MSNs expressing the D1-receptor, we stained for Atf3 after chronic L-DOPA stimulation (4 hours timepoint after the last L-DOPA administration) as shown in Figure S6D.

Overall, these data suggested that increased transcription from a subset of PcG-repressed genes was associated with the development of LID. The expression level of H3K27me3 and H3K27me3S28p marked genes was generally lower in chronic L-DOPA stimulated mice compared to acute L-DOPA stimulated mice, correlating with reduced induction of the H3K27me3S28p mark. However, repeated L-DOPA administration resulted in the induction of a unique group of PcG regulated genes that were not induced after a single L-DOPA injection. For the group of genes that gained H3K27me3S28p (subgroup II in Figure 6B) and had significantly higher expression in chronic L-DOPA stimulated lesioned mice compared to acute stimulated mice the most significantly enriched GO-term was “*Adult behavior*”. However, the term comprises only five genes from subpopulation II (Figure 6B): *Nr4a2*, *Nr4a3*, *Trh*, *Npy* and *Adra1b* (out of 93 potential genes in the genome), and although this was a significant (p = 0.0011) 10.7 fold enrichment over the expected number, it did not remain significant when Benjamini-Hockberg corrected for multiple testing.

## Discussion

### *In vivo* evidence for a functional role of H3K27me3S28 phosphorylation in gene regulation

We have previously shown that H3K27me3S28 phosphorylation causes the displacement of PRC1- and PRC2-complexes from chromatin, leading to expression of a subset of PcG regulated genes in cultured human fibroblasts [18]. Here, we for the first time provide *in vivo* evidence for the relevance of this mechanism for gene regulation in adult, post-mitotic neurons utilizing a mouse model of PD. We show by genome wide analyses that, in striatal MSNs, signaling through sensitized D1Rs induces H3S28 phosphorylation in the context of H3K27me3 marked genes. Importantly, the H3K27me3S28p mark correlates with a reduction in PcG binding and increased transcription of a subset of genes, several of which have been implicated in neuronal plasticity.

Notably, a systemic injection of a specific D1R agonist was also able to induce the H3K27me3S28p mark in naïve mice. This clearly indicates that the mechanisms described in this study have a general relevance with regard to D1R transmission in the adult brain.

### Genome wide analyses of H3K27me3S28 phosphorylation and transcriptional consequences

Taking advantage of the pronounced effects produced by dopamine depletion, we mapped putative downstream genomic targets regulated by L-DOPA via D1Rs. Our genome-wide analysis showed that at least 1/3 of all H3K27me3 marked gene loci, that can potentially give rise to a mRNA transcript in the non-repressed state, gained H3K27me3S28 phosphorylation upon acute L-DOPA administration. Most importantly, among regulated transcripts, phosphorylation of S28 in the context of H3K27me3 was a strong indicator of transcriptional activation.

Interestingly, the combined analyses of RNA-seq and ChIP-seq data showed that the majority of transcripts originating from H3K27me3 genomic loci that gained H3K27me3S28 phosphorylation did not change expression upon L-DOPA stimulation. As previously demonstrated, H3K27me3S28 phosphorylation leads to removal of PcG complexes at H3K27me3 marked regions and is considered to derepress the promoter [18]. Therefore it appears that, in order to fully activate genes, H3K27me3S28 phosphorylation requires additional events to take place, which likely would be H3K4 methylation and histone acetylation.

### Msk1 and PP1 influence the overall level of H3K27me3S28p on target genes

In line with our previous findings, induction of the H3K27me3S28p mark in response to L-DOPA was reduced in 6-OHDA lesioned Msk1−/− mice compared to wt mice (Figure S5). We found that the induction of H3K27me3S28p was less pronounced, but not abolished, in Msk1−/− mice, indicating that in striatal MSNs other histone kinases must be present and actively relay dopamine signaling to chromatin.

**Figure 7:**
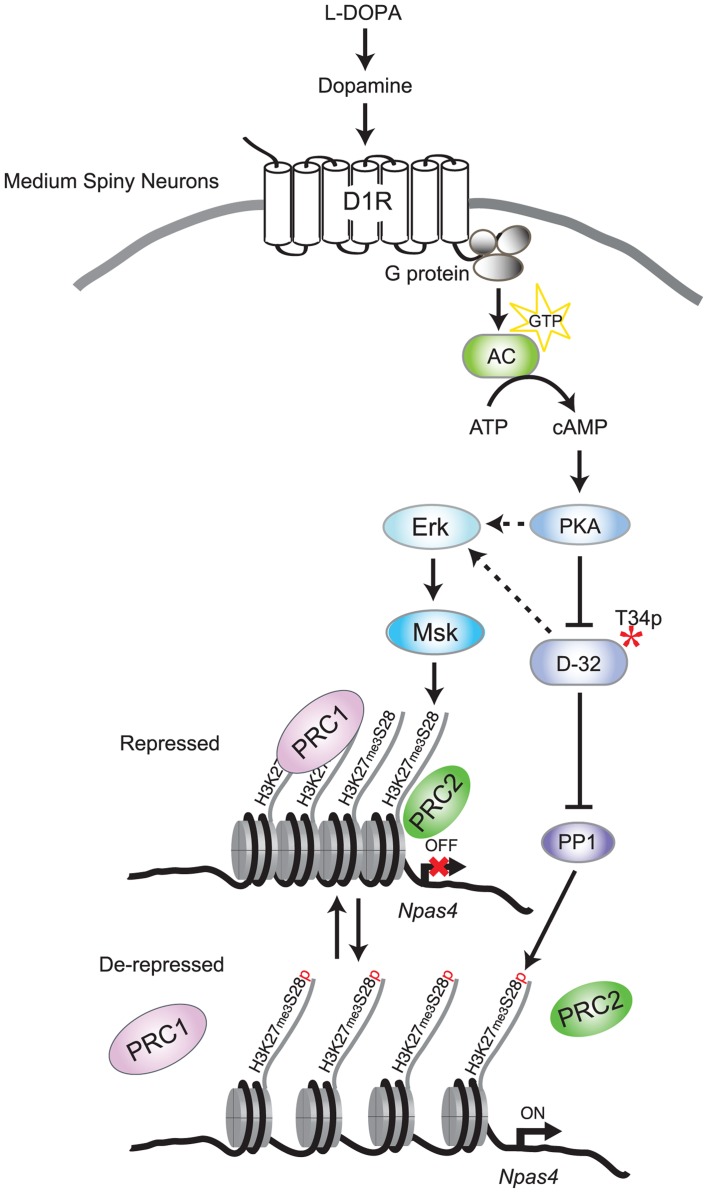
Model for L-DOPA induced signaling and H3K27me3S28 phosphorylation in MSNs of parkisonian mice. In 6-OHDA lesioned mice, L-DOPA is converted to dopamine which binds to sensitized D1Rs. Activation of D1Rs stimulates adenylate cyclase (AC), leading to increased cAMP and activation of PKA/DARPP-32 signaling, which promotes Erk phosphorylation. Erk activates Msk leading to H3K27me3S28 phosphorylation. In addition, PKA-mediated activation of DARPP-32 inhibits PP1, further increasing H3K27me3S28 phosphorylation. Augmented H3K27me3S28p leads to reduced binding of PcG complexes (PRC1 and PRC2), derepression of genes and eventually increased transcription depending on general transcription factors and RNA polymerase II (not included in this illustration for simplicity).

The phosphatase responsible for the dephosphorylation of H3K27me3S28p has so far been elusive. Our results, showing that H3K27me3S28 phosphorylation induced by L-DOPA is decreased by abolishing PKA-mediated activation of DARPP-32, pointed to PP1 as a plausible candidate. This idea was corroborated by *in vitro* and *ex vivo* experiments showing that PP1 could dephosphorylate H3K27me3S28p (Figure 5B and C). Overall, our results support a model in which inhibition of PP1 via PKA/DARPP-32 works in parallel with Msk1 to promote H3K27me3S28 phosphorylation in response to activation of D1R (see model in Figure 7).

### Deregulation of PcG bound genes through H3K27me3S28 phosphorylation and its potential role in dyskinesia

Dyskinesia is a serious motor complication caused by prolonged administration of L-DOPA to parkinsonian patients [9]. It has been proposed that LID depends on the persistent and intermittent hyper-activation of the cAMP/DARPP-32 signaling cascade produced by L-DOPA through sensitized D1Rs. This, in turn, leads to hyper-phosphorylation of histone H3 and aberrant expression of specific genes implicated in dyskinetic behavior [10], [11]. We have previously shown that a T34A mutation on DARPP-32 decreases dyskinetic behaviour in 6-OHDA lesioned mice and that this effect correlates with reduced histone H3S10 phosphorylation [14]. In this study, we found that the induction produced by L-DOPA on H3K27me3S28 phosphorylation at regulatory regions of the *Atf3* and *Npas4* genes, which are bound and repressed by PcG proteins in MSNs under normal conditions, correlated with increased mRNA- and protein synthesis. We also show that H3K27me3S28 phosphorylation and the associated transcriptional activation are largely reduced in DARPP-32 T34A mutant mice. These observations are particularly interesting in view of the involvement of *Atf3* and *Npas4* in synaptic plasticity and long-term adaptive responses. Augmented expression of Atf3 in the dorsal striatum has been observed following acute and chronic administration of amphetamine [32], a drug that, similarly to L-DOPA, promotes dopamine transmission. Furthermore, the TF Npas4 has been proposed to promote the expression of other immediate early genes, including *Arc*, *Egr1* and *c-Fos* [33], which have all been associated to chronic administration of L-DOPA and to the development of LID [15], [34], [35].

Our results show that repeated administration of L-DOPA decreases its ability to induce global phosphorylation of H3K27me3S28 (Figure 6A). This is not surprising since desensitization of kinase signaling pathways following persistent upstream activation and negative feedback is a common phenomenon. Accordingly, previous work showed that repeated administration of L-DOPA to 6-OHDA-lesioned mice is accompanied by a partial normalization of sensitized D1R signaling, reflected in lower levels of Erk activation and reduced global phosphorylation of H3 at S10 [15], [36], [37]. Importantly, despite the attenuated induction of H3K27me3S28 phosphorylation after chronic L-DOPA, our genome-wide analysis showed that there was still a good correlation between the genomic loci gaining the H3K27me3S28p mark and gene induction. This suggested that residual kinase activity was sufficient to drive transcription from PcG-repressed genes in the context of LID (Figure S6A–C).

The decreased ability of L-DOPA to induce H3K27me3S28p observed after chronic administration correlated with reduced expression of transcripts from H3K27me3 and H3K27me3S28p marked genes (Figure 6B), but not of transcripts from H3K27me3 marked genes lacking the H3K27me3S28p mark or transcripts in general (Figure 6C–D). As an exception, IE genes such as *Atf3*, *c-Fos* and *Egr2-4*, which encode TFs, were all induced to at least the same extent after chronic L-DOPA administration compared to acute administration (Table S2). This suggests that these IE genes, which are characterized by transient expression, respond with fast on/off-kinetics presumably due to rapid dephosphorylation of S28 by PP1. This would allow PcG complexes to re-bind, when the signal leading to Msk1 activation ceases, thereby re-setting gene repression until the next activating signal, like for the repeated daily L-DOPA administration in chronically treated parkisonian mice.

The IE genes, *Nr4a2* and *Nr4a3*, also known as *Nurr1* and *Nor1*, encode orphan nuclear receptors that function as TFs. These genes were more strongly induced after repeated L-DOPA administration compared to acute L-DOPA administration. It has been shown previously that the expression of Nr4a2, a gene product involved in the development of dopaminergic neurons [38], is increased in response to prolonged treatment with L-DOPA and that this effect occurs in the MSNs expressing D1Rs [39]. Notably, recent evidence indicates that viral vector-induced overexpression of Nr4a2 in striatal neurons increases dyskinesia in a rat model of PD [40]. Our results are in line with these observations and provide a possible mechanism accounting for the enhanced expression of Nr4a2 in response to chronic L-DOPA administration. In the striatum, Nr4a3 mRNA expression is induced by activation of D1Rs and this effect is prevented by blockade of Erk signaling [41]. Whereas the exact role of Nr4a3 in dopaminergic transmission remains to be elucidated, the present data support the idea that, in PD, this gene is de-repressed via D1R-mediated activation of Erk and increased H3K27me3S28 phosphorylation.

Interestingly, we identified other PcG regulated genes such as neuropeptide encoding genes: galanin (*Gal*), thyrotropin releasing hormone (*Trh*) and neuropeptide Y (*Npy*), which all were only induced upon chronic treatment with L-DOPA (Table S2). In contrast to IE genes, these neuropeptide encoding genes seems to be more tightly regulated, and their activation presumably requires persistent phosphorylation of H3K27me3 marked and PcG bound regions, as well as induction of additional TFs and co-factors to lead to their transcriptional activation.

The regulation of Trh is in line with previous work showing a large increase in the mRNA for this hormone occurring in the striatal MSNs of dyskinetic rats [42]. Subsequent analysis established a correlation between Trh expression in D1R-expressing MSNs and dosage of L-DOPA, which is regarded as a critical factor in the development of dyskinesia [39]. Indeed, enhanced levels of Trh may concur to the development of dyskinesia, since hyperthyroidism is typically associated to hyperkinesia.

Increased levels of NPY mRNA have been found in the striata of Parkinsonian patients treated with L-DOPA [43]. The present data suggest the possibility that this increase may occur in striatal MSNs, although in naïve mice, NPY is mainly expressed in GABAergic interneurons [44]. NPY has been proposed to exert neuroprotective effects on dopaminergic neurons [45], however further studies will be necessary to clarify its potential role in dyskinesia.

Galanin (Gal) and galanin receptors are involved in many neuronal functions including drug addiction [46]. In the striatum, galanin receptors have been localized to cholinergic interneurons and neuronal terminals, which are critically involved in the regulation of the excitability of MSNs [47], [48]. Galanin has also been shown to reduce dopamine release in the striatum [49] and to inhibit spontaneous locomotion by reducing the activity of midbrain dopaminergic neurons [50]. Therefore, the increase in galanin mRNA expression produced by chronic administration of L-DOPA may lead to significant modifications in basal ganglia neurotransmission. Future studies will be necessary to determine the impact of these changes on the development and manifestation of dyskinesia.

The functional implications of differences in reactivation of PcG repressed genes remain to be investigated in more detail. However, it is possible that genes that are activated only in response to acute administration of L-DOPA are implicated specifically in the emergence of LID, whereas genes that are activated only in response to chronic L-DOPA are involved in the consolidation and manifestation of this condition. Interestingly, one of the receptors for the neuropeptide galanin, Galr2 was only induced in response to acute L-DOPA (Table S1 and Figure 6F), while the gene encoding the ligand, Gal, was only induced by chronic L-DOPA stimulation as mentioned above.

Further studies are necessary to examine which permissive determinants are required at genomic sites for H3K27me3S28p mediated expression of PcG target genes. Nevertheless, the fact that many genes regulated by PcG proteins are TFs and among them many are affected by H3K27me3S28 phosphorylation and upregulated by acute and chronic L-DOPA, suggest that LID is, at least in part, a consequence of secondary transcriptional events. These TFs might only need to be induced transiently in order to trigger transcription of other genes that lead to sustained changes in neuronal plasticity associated to long-term maladaptive responses, such as dyskinesia.

Overall our novel findings reveal a previously unrecognized plasticity of PcG-repressed genes in terminally differentiated neurons. The identification of specific genes whose expression is increased upon prolonged treatment with L-DOPA and dopamine D1 receptor stimulation offer a possibility to design novel therapeutic strategies to treat Parkinson's disease and potentially other disorders caused by dysfunctional dopaminergic transmission in the brain, such as drug addiction and schizophrenia.

## Materials and Methods

### Animals

Female C57BL/6J mice (30 g) were purchased from Taconic (Tornbjerg, Denmark). Bacterial artificial chromosomes transgenic mice expressing EGFP under the control of the promoter for the D2R (*Drd2*-EGFP) or the dopamine D1R (*Drd1a*-EGFP) were generated by the Gene Expression Nervous System Atlas program at the Rockefeller University [27] and were crossed on a C57BL/6 background for three generations. DARPP-32 T34A mutant mice [51] and Msk1−/− mice [52] have already been described. The animals were housed in groups of five under standardized conditions with 12 hours light/dark cycle, stable temperature (20°C), and humidity (40–50%). All protocols utilized to generate the model of PD and dyskinesia (including chronic administration of L-DOPA), were approved by the Research Ethics Committee of Karolinska Institutet and by the Swedish Animal Welfare Agency (permit N515/12).

### 6-OHDA lesion

Mice were anesthetized with a mixture of fentanyl citrate (0.315 mg/ml), fluanisone (10 mg/ml) (VetaPharma, Leeds, UK), midazolam (5 mg/ml) (Hameln Pharmaceuticals, Gloucester, UK), and water (1∶1∶2 in a volume of 10 ml/kg) and mounted in a stereotaxic frame (David Kopf Instruments, Tujunga, CA) equipped with a mouse adaptor. 6-OHDA-HCl (Sigma-Aldrich Sweden AB) was dissolved in 0.02% ascorbic acid in saline at a concentration of 3 µg of free-base 6-OHDA per microliter. Each mouse received one unilateral (right hemisphere) injection of 6-OHDA of 1 µl (0.5 µl/min) into the medial forebrain bundle according to the following coordinates (mm): anteroposterior (AP), −1.2; mediolateral (ML), −1.2; dorsoventral (DV), −4.8 (all millimeters relative to bregma) [53]. Noradrenergic neurons were protected by injection of 25 mg/kg desipramine (Sigma) thirty minutes prior to 6-OHDA injection. This procedure leads to a decrease in striatal tyrosine hydroxylase immunoreactivity ≥80% and to a marked akinesia affecting the side of the body contralateral to the lesioned striatum. Animals were allowed to recover for 2 weeks before experimentation.

### Mouse treatment

L-DOPA (10 mg/kg in combination with 7.5 mg/kg benserazide; purchased from Sigma) was dissolved in saline (0.9% NaCl), and injected intraperitoneally in a volume of 10 ml per kilogram of body weight for 1, 3 or 9 days. SKF81297 (3 mg/kg) was purchased from Tocris.

### Cell culture experiments

Mouse embryonic stem (mES) cells, wildtype (wt) E14 (provided by Dr. Zhou-Feng Chen and Dr. Helle Færk Jørgensen) and Eed^−/−^ (provided by Dr. Anton Wutz) were cultured on 0.1% (w/v) gelatin-coated plates in ES medium (Glasgow Minimum Essential Medium (Sigma) supplemented with Glutamax-1 (Gibco), non-essential amino acids (Gibco), 50 mM 2-mercaptoethanol, 15% (v/v) ES-cell-qualified FBS (Gibco), and 1% (v/v) penicillin/streptomycin) in the presence of 1,000 U/ml of LIF (Millipore). To induce histone phosphorylation, the mES cells were stimulated with 1 µg/mL anisomycin in DMSO or DMSO only as control.

For ChIP, cells were cross-linked for 10 min at room temperature in culture media containing 1% formaldehyde, 10 mM Hepes (pH 8.0), 0.1 mM EGTA, and 20 mM NaCl. Cross-linking was stopped by addition of glycine to a final concentration of 0.125 M, followed by an additional incubation for 5 min. Fixed cells were washed 3 times with PBS and harvested in SDS lysis buffer (50 mM Tris at pH 8.1, 0.5% SDS, 100 mM NaCl, 5 mM EDTA, 1 mM PMSF, 10 µg/ml leupeptin and 10 µg/ml aprotinin). The cells were then pelleted for 10 min at 2,400 g followed by the same ChIP protocol as for striatal tissue. The included primer sequences are listed in Table S3.

### Western blotting

Mice were killed by decapitation, the heads of the animals were cooled in liquid nitrogen for 6 s and the brains were removed. Coronal slices of 1 mm thickness were obtained from a mouse brain dissection matrix (Activational Systems Inc., RBM-2000C), and three striatal punches of 2 mm diameter from sequential slices were dissected out on an ice-cold surface, sonicated in 1% SDS, and boiled for 10 min. Proteins were separated by SDS–polyacrylamide gel electrophoresis and transferred overnight to PVDF membranes (Amersham Pharmacia Biotech, Uppsala, Sweden). The following antibodies were used: H3S28p (Millipore, 07-145), H3K27me3S28p (Hansen lab), histone H3 (Abcam, ab1791), Atf3 (Santa Cruz, sc-188). The Npas4 antibody was provided by Prof. Greenberg, Harvard Medical School, Boston, USA.

### Immunohistochemistry

Mice were rapidly anaesthetized with pentobarbital (300 mg/kg ip, Sanofi-Aventis, France) and perfused transcardially with 4% (w/v) paraformaldehyde in 0.1 M sodium phosphate buffer (pH 7.5). Brains were post-fixed overnight in the same solution and stored at 4°C. Forty-micrometer-thick sections were cut with a vibratome (Leica, Nussloch, Germany). Free-floating sections were rinsed in tris-buffered saline, permeabilized in 0.2% Triton X-100 in TBS for 20 min and blocked to prevent non-specific binding by incubation in 0.5% Triton X-100, 5% normal goat serum, 1% bovine serum albumin in TBS for 1 hr at RT. Sections were incubated overnight at 4°C with primary antibodies. The following antibodies were used: EGFP (Aves Lab, GFP-1020), NeuN (Abcam, ab138452). Antibodies for histone marks were the same as for Western blotting. Images from the dorsolateral striatum were obtained by sequential laser scanning confocal microscopy (Zeiss LSM 510 Meta). Data was analyzed by one-way or two-way ANOVA when appropriate followed by Tukey's HSD post-hoc test. Unpaired t-test was used when comparing two means. p<0.05 was considered significant.

### *Ex vivo* slice experiments

Naive C57BL/6 mice were killed by decapitation, and the brains were rapidly removed. Coronal slices (250 µm) were prepared with the use of a vibratome (Leica, Nussloch, Germany). Dorsal striata were dissected out from each slice under a microscope. Two slices were placed in individual 5-ml polypropylene tubes containing 2 ml of Krebs-Ringer bicarbonate buffer. The samples were equilibrated at 30°C for 30 minutes, followed by incubation of the slices with either vehicle (DMSO), 100 nM or 1 µM okadaic acid (Sigma) in 2 mL fresh buffer for 50 min. After incubation, the solutions were rapidly removed, the slices were sonicated in 1% SDS, and the samples were analyzed by Western blotting as described.

### *In vitro* Protein Phosphatase 1 (PP1) dephosphorylation assay using histone peptides

Ten µg of N-terminal H3 peptides representing the first 40 amino acids of histone H3.1 were used per reaction, either unmodified or modified as follows: S28 phosphorylated (H3S28p) or K27 tri-methylated and S28 phosphorylated (H3K27me3S28p). PP1 (1 unit) was added to the reaction containing 10 µg of specified H3 peptide in a 15 µl de-phosphorylation buffer: 50 mM HEPES, pH 7.5, 100 mM NaCl, 2 mM DTT, 0.01% Brij 35 and 1 mM MnCl_2_. The de-phosphorylation reaction was allowed to proceed at 30°C, for 30 min and stopped by adding EDTA (pH 8.0) to a final concentration of 5 mM. A fraction of each reaction was spotted on a nitrocellulose membrane corresponding to: 1.0 µg, 0.1 µg and 0.01 µg H3.1 peptide. The membrane was blocked as for standard Western blotting and developed using antibodies for H3S28p (Millipore 07-145, 1∶3,000) and secondary anti-rabbit HRP (Vector Laboratories). Dot-blots were added enhanced chemiluminiscence (ECL) after the last wash and exposures were made using a ImageQuant LAS 4000 camera system. The quantifications were made based on the dots containing 1 µg peptide.

### Chromatin immunoprecipitation (ChIP)

Tissue punches for chromatin preparation was obtained as described for Western blotting. The punches were fixed for 12 min in cold 1% formaldehyde/PBS followed by glycine incubation to stop further cross-linking. The fixed punches were then washed 3× with cold PBS containing phosphatase inhibitors (20 nM okadaic acid, 10 µM NaF) and subsequently snap-frozen for later use. Chromatin immunoprecipitation experiments were performed as described [54] with some modifications: Fixed striatal punches were homogenized in a nuclear extraction buffer (10 mM Tris (pH 8.0), 100 mM NaCl, 2 mM MgCl_2_, 0.3 M Sucrose, 0.25% IGEPAL CA-630) containing protease inhibitors (1 mM PMSF, 0.1 mM aprotinin, 0.1 mM leupeptin) and phosphatase inhibitors (20 nM okadaic acid, 10 µM NaF), by douncing 15 times using a 2 mL loose grind pestle followed by a 30 min incubation on ice. The homogenate was dounced another 50 times using a 2 mL loose grind pestle for nuclear release, followed by 10 min centrifugation at 2,400 g to pellet nuclei. The extracted nuclei were then lysed in a lysis buffer containing 50 mM Tris-HCl (pH 8.0), 10 mM EDTA, 1% (wt/vol) SDS and protease/phosphatase inhibitors, diluted in RIPA buffer (10 mM Tris-HCl (pH 7.5), 140 mM NaCl, 1 mM EDTA, 0.5 mM EGTA, 0.1% (vol/vol) Triton-X-100, 0.1% (wt/vol) SDS, 0.1% (wt/vol) Na-deoxycholate) and the DNA was sonicated to an average size of 300–500 bp using a Bioruptor standard device (Diagenode) (10 cycles 30 sec ON, 30 sec OFF, highest setting).

10 µg anti-rabbit IgG (DAKO), 3 µg of anti-H3 (“GERA”, Hansen lab), 2.5 µg of anti-Rnf2 (“NAST”, Hansen lab) anti-H3K27me3 (9756, Cell Signaling) and 2 µg anti-H3K4me3 (Lys4) (9751, Cell Signaling) and anti-H3K27me3S28p (the specificity of the batch #5 of peptide antigen purified H3K27me3S28p antibody used in this study was tested as shown in Figure S2) was incubated at 4°C with 25 µL washed Dynabeads protein A (Invitrogen) and RIPA in a total volume of 100 µL. The bead-antibody complexes were then incubated at 4°C for 2 h with 20 µL chromatin in a total volume of 250 µL. Beads were washed in 3× RIPA, 1× high salt wash buffer (20 mM Tris-HCl (pH 7.5), 500 mM NaCl, 2 mM EDTA, 0.1% Triton-X-100, 0.1% SDS), 1× LiCl buffer (10 mM Tris-HCl (pH 7.5), 250 mM LiCl, 1 mM EDTA, 1% Na-deoxycholate, 1% IGEPAL CA-630) and 1× TE buffer. After washes, DNA was eluted from beads and de-crosslinked in 20 mM Tris-HCl, pH 7.5, 5 mM EDTA, 50 mM NaCl. 1% (wt/vol) SDS and 50 µg/mL protease K at 68°C overnight. For input, 20 µL chromatin was de-crosslinked in 20 mM Tris-HCl, pH 7.5, 5 mM EDTA, 50 mM NaCl 68°C overnight. ChIP and input DNA was then purified and eluted using Minelute PCR purification kit (Qiagen). Enrichments on selected loci were measured by qPCR, 3 technical replicates, (7500 Fast, Applied Biosystems) relative to a 5-point dilution series of input chromatin. The included primer sequences are listed in Table S3. Student's *t* test were used to compare means of the different conditions.

### ChIP-sequencing

ChIPs for each experimental condition was performed in at least triplicates. The resulting immunoprecipitated DNA were pooled and prepared for ChIP sequencing using an Illumina kit according to the manufacturer's guidelines. 2 nanogram of starting material, as determined by PicoGreen concentrations, was used in each case. Sequencing was performed on a Genome Analyzer II (Illumina) at the National High-throughput Sequencing Centre in Copenhagen.

Libraries were de-multiplexed and high quality reads (Chastity score > = 0.6) were aligned to the mouse genome (mm9) using Bowtie [55] allowing up to two mismatches. Reads not aligning uniquely to the mouse genome were removed and only unique reads were used for subsequent analysis. Tracks from single genomic loci were presented using the UCSC Genome Browser (http://genome.ucsc.edu/) [56]. Reads were normalized to a library size of 10M reads and converted to wig-files using the program EaSeq (Lerdrup et al, manuscript in preparation).

### ChIP-sequencing analysis

All quantitation, scoring, gating, and visualization was done in the program EaSeq (Lerdrup et al, manuscript in preparation). A list of all transcripts including genomic coordinates was derived from Genomatix (see RNA-sequencing for details), and the amounts of ChIP-seq signal at the genomic regions corresponding to −1 kbp to +1 bkp was quantified and normalized to dataset size and a region-size of 1 kbp. Transcripts were scored positive or negative by fitting the abundance to the quantified ChIP-seq signal at all transcripts genome-wide to a normal distribution. A threshold was automatically applied at the level where the only 5% of the regions were expected to score positive by chance (thresholds were 6.8 for left hemisphere H3K27me3 and 16.9 for right hemisphere H3K27me3S28P). Transcripts were gated into subpopulations depending on the level of ChIP-seq signal relative to these thresholds and/or fold change in gene expression as well as significance in Benjamini-Hochberg corrected p-values [57].

### Gene expression analysis

One striatal punch per hemisphere was dissected out and subsequently put in RNAlater (Qiagen) at +4°C over night to inhibit RNA degradation. Total RNA was extracted using a RNeasy kit (Qiagen) and quantified on a NanoDrop 1000 device. 200 ng of RNA was used for generation of cDNA using a TaqMan Reverse Transcription Reagents kit (Invitrogen). Expression levels for individual transcripts were measured by qPCR and calculated by the ddCt-method using TATA-binding protein (*Tbp*) mRNA as housekeeping gene. The expression levels were based on 3 biological replicates. The included primer sequences are listed in Table S3.

### RNA-sequencing

A TruSeq RNA Sample preparation kit (Illumina) was used for library generation out of 0.5 µg of total RNA per condition. The generated libraries were sequenced on a Genome Analyzer II (Illumina) at the National High-throughput Sequencing Centre in Copenhagen. Reads were mapped to the mouse genome (mm9) using the Genomatix Mining Station software (Genomatix). Differential expression analysis was done on triplicates using the region miner task “Expression Analysis for RNASeq Data” on a Genomatix Genome Analyzer (Genomatix) using the DeSeq algorithm [58].

### Gene ontology analysis

Gene ontology (GO) enrichment analyses were done from sets of genes with a significant (Benjanimi-Hochberg corrected for multiple testing) increase or decrease in expression of at least 1.5 fold using the DAVID functional annotation tool at http://david.abcc.ncifcrf.gov/ [59], [60].

### Accession number

The Geo accession number for the ChIP-seq and RNA-seq data reported in this paper is GSE60703.

## Supporting Information

Figure S1 The H3K27me3S28p mark is induced in the striatum after injection of a specific dopamine D1-receptor agonist. A. Representative confocal micrographs of H3K27me3S28p immunoreactivity. Acute SKF81297 administration (1 hour) increased striatal H3K27me3S28 phosphorylation in naïve animals compared to vehicle-treated animals (t_(4)_ = 5.22, ** p<0.01). n = 3 per condition. Data are means ± S.E.M. * p<0.05, ** p<0.01, *** p<0.001. B. Lesioned (6-OHDA treated) and unlesioned mice (vehicle treated) were treated chronically with L-DOPA (every day for 9 days) and proteins were extracted from tissue samples 1 hour after the last L-DOPA injection and subjected to Western blotting using antibodies for H3K27me3S28p and general H3. The H3K27me3S28p signal was normalized to H3 and the ratio was plotted in the lower part as arbitrary units. Data are means ± S.E.M. from three independent experiments. *** p<0.001.
(EPS)

Figure S2 Validation of the H3K27me3S28p antibody. A. Dot-blot analysis using synthetic peptides representing the N-terminal tail of histone H3.1, with the indicated chemical modifications, that were spotted in amounts as shown on the right hand side of the membrane. The membrane was incubated with H3K27me3S28p antibody (batch #5) and processed as described in the Suppl. Materials and Methods section. B. ChIP-qPCR on chromatin from wt and Eed−/− mouse ES cells after 1 h stimulation with anisomycin or DMSO. Antibodies for H3K27me3, H3K27me3S28p, and primers for regulatory regions of *Jun and Egr1* were used. Signals from histone marks are normalized to total histone H3 signals. Data are means ± S.E.M of triplicate qPCR reactions. The statistical significance of the signal in the anisomycin treated cells is compared to the respective signal in the DMSO treated cells, * p<0.05, ** p<0.01, *** p<0.001.
(EPS)

Figure S3 Representative fluorescence micrographs of NeuN immunoreactivity (red) and DAPI (blue). Approximately 90.0±3.7% out of a total of 2,686 counted nuclei were neurons as they positively stained for the pan-neuronal marker NeuN+. Data is mean ± S.E.M.
(EPS)

Figure S4 GO-analysis. GO-terms of significantly up-regulated H3K27me3 and H3K27me3S28p positive transcripts in the sub-populations indicated with roman numerals in Figure 3G–I. Values on the axis are −log(p-values) of the most significant GO-terms (Benjamini-Hochberg corrected for multiple testing). Red coloring covers the values below 0.05 (∼−1.3 when log10 transformed).
(EPS)

Figure S5 Attenuated H3K27me3S28 phosphorylation in Msk1 KO mice. H3K27me3S28 phosphorylation was attenuated in Msk1−/− mice following acute administration of L-DOPA (lesion x genotype interaction: F_(1,16)_ = 9.76, p = 0.007). *** p<0.001 vs unlesioned hemisphere of the same genotype, °° p<0.01 vs lesioned wt hemisphere. n = 5 per condition. Data are means ± S.E.M. * p<0.05, ** p<0.01, *** p<0.001.
(EPS)

Figure S6 Histograms of the fold change in expression in lesioned striata compared to unlesioned striata after chronic L-DOPA of transcripts from H3K27me3S28p positive and H3K27me3 positive genes (A), from H3K27me3S28p-negative but H3K27me3-positive genes (B), and from genes regardless of specific histone marks (C). D. Immunostaining for Atf3 (red) in unlesioned and lesioned striata after 9 days of chronic L-DOPA administration (4 hours timepoint after last L-DOPA administration) of D1-EGFP (green) expressing mice. Scale bar 10 µm.
(PDF)

Figure S7 ChIP-seq signals for input chromatin, H3K4me3, H3K27me3 and H3K27me3S28p in unlesioned and lesioned striata 1 hour after injection of L-DOPA (acute) in unilaterally 6-OHDA lesioned mice, across genomic regions near the A) *Ppm1n* and *Galr2* genes and B) near the *Nr4a2* (also known as *Nurr1*), *Ngf* and *Lipg* genes.
(EPS)

Table S1 List of unlesioned striatum H3K27me3 and lesioned striatum H3K27me3S28p positive genes with a significant (p>0.05) fold change of more than log2 = 0.58 in the lesioned hemisphere compared to the unlesioned hemisphere 1 h after a first L-DOPA injection (“acute”) based on RNA-seq data. The corresponding change in expression in chronically L-DOPA treated lesioned to unlesioned hemispheres are listed in the second column if significant. Genes with a normalized expression value of <0.004 were considered non- detectable. Genes that significantly changed expression in the unlesioned striata after L-DOPA injection and genes that changed expression in the lesioned hemisphere compared to the unlesioned hemisphere were not listed.
(EPS)

Table S2 List of unlesioned striatum H3K27me3 and lesioned striatum H3K27me3S28p positive genes with a significant (p>0.05) fold change of more than log2 = 0.58 in the lesioned hemisphere compared to the unlesioned hemisphere 1 h after a last L-DOPA injection after 9 days of daily L-DOPA injections (“chronic”) based on RNA-seq data. The corresponding change in expression in acutely L-DOPA treated lesioned to unlesioned hemispheres are listed in the second column if significant. Genes with a normalized expression value of <0.004 were considered non-detectable. Genes that significantly changed expression in the unlesioned striata after L-DOPA injection and genes that changed expression in the lesioned hemisphere compared to the unlesioned hemisphere were not listed.
(EPS)

Table S3 List of primers for ChIP-qPCR and RT-qPCR analyses used throughout this study.
(EPS)

Text S1 Describes supplementary methods for additional cell culture experiments, dot-blot analysis and immunohistochemistry.
(DOCX)
