# Supplementary figures and images for: Dopamine Signaling Leads to Loss of Polycomb Repression and Aberrant Gene Activation in Experimental Parkinsonism
Source: PLoS Genet. 2014 Sep 25;10(9):e1004574. doi: 10.1371/journal.pgen.1004574 (PMC4177666; doi:10.1371/journal.pgen.1004574)

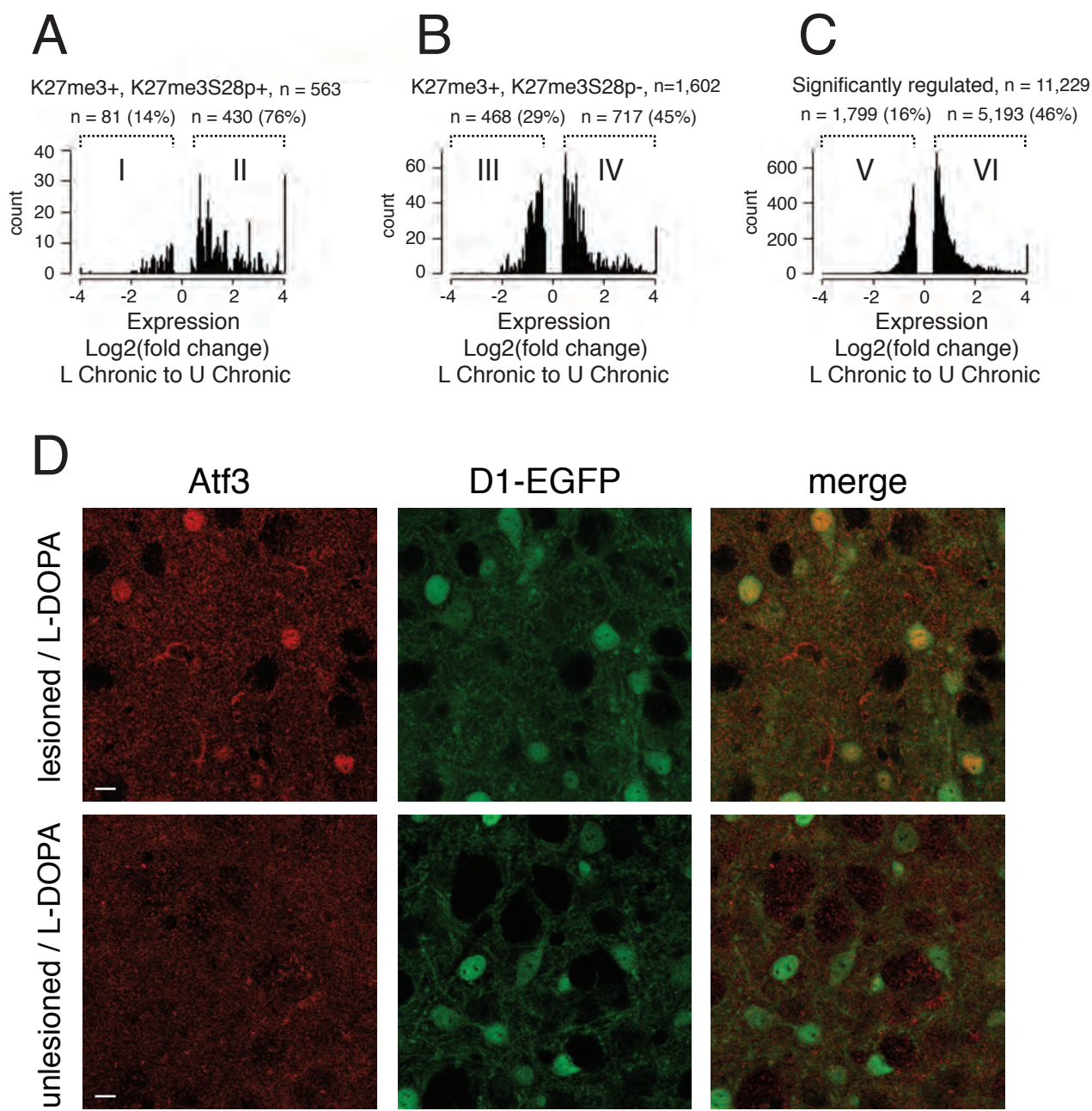

Supplement: Figure S6 — Histograms of the fold change in expression in lesioned striata compared to unlesioned striata after chronic L-DOPA of transcripts from H3K27me3S28p positive and H3K27me3 positive genes (A), from H3K27me3S28p-negative but H3K27me3-positive genes (B), and from genes regardless of specific histone marks (C). D. Immunostaining for Atf3 (red) in unlesioned and lesioned striata after 9 days of chronic L-DOPA administration (4 hours timepoint after last L-DOPA administration) of D1-EGFP (green) expressing mice. Scale bar 10 µm. (PDF) [file pgen.1004574.s006.pdf]
