# Supplementary material for: Dopamine Signaling Leads to Loss of Polycomb Repression and Aberrant Gene Activation in Experimental Parkinsonism
Source: PLoS Genet. 2014 Sep 25;10(9):e1004574. doi: 10.1371/journal.pgen.1004574 (PMC4177666; doi:10.1371/journal.pgen.1004574)
Supplement: Text S1 — Describes supplementary methods for additional cell culture experiments, dot-blot analysis and immunohistochemistry. (DOCX) [file pgen.1004574.s011.docx]

**TEXT S1**

**SUPPLEMENTARY METHODS**

**Cell culture experiments**

Mouse embryonic stem (mES) cells, wildtype (wt) E14 (provided by Dr. Zhou-Feng Chen and Dr. Helle Færk Jørgensen) and Eed^-/-^ (provided by Dr. Anton Wutz) were cultured on 0.1% (w/v) gelatin-coated plates in ES medium (Glasgow Minimum Essential Medium (Sigma) supplemented with Glutamax-1 (Gibco), non-essential amino acids (Gibco), 50 mM 2-mercaptoethanol, 15% (v/v) ES-cell-qualified FBS (Gibco), and 1% (v/v) penicillin/streptomycin) in the presence of 1,000 U/ml of LIF (Millipore). To induce histone phosphorylation, the mES cells were stimulated with 1μg/mL anisomycin in DMSO or DMSO only as control.

For ChIP, cells were cross-linked for 10 min at room temperature in culture media containing 1% formaldehyde, 10 mM Hepes (pH 8.0), 0.1 mM EGTA, and 20 mM NaCl. Cross-linking was stopped by addition of glycine to a final concentration of 0.125 M, followed by an additional incubation for 5 min. Fixed cells were washed 3 times with PBS and harvested in SDS lysis buffer (50 mM Tris at pH 8.1, 0.5% SDS, 100 mM NaCl, 5 mM EDTA, 1 mM PMSF, 10 µg/ml leupeptin and 10 µg/ml aprotinin). The cells were then pelleted for 10 min at 2,400 g followed by the same ChIP protocol as for striatal tissue. The included primer sequences are listed in Suppl. Table S3.

**Dot-blot analysis**

Peptides representing the N-terminal 40 aa´s of human histone H3.1 with the following modifications: H3K27me3: tri-methylation of lysine 27 (K27); H3S28p: phosphorylation of serine 28 (S28); H3K27me3S28p: tri-methylated at lysine 27 (K27) and phosphorylated at serine 28 (S28); H3K9me3S10p: tri-methylated at lysine 9 (K9) and phosphorylated at serine 10 (S10). The peptides were spotted onto a nitrocellulose membrane (Hybond C-extra) in amounts corresponding to 1.000 µg, 0.100 µg, 0.010 µg and 0.001 µg. The membrane was blocked followed by incubation with H3K27me3S28p antibody (batch #5) for 2 hours, washed and incubated with anti-rabbit HRP antibody (1:5,000 dilution). After 45 min incubation the membrane was washed and processed for development using enhanced chemiluminescence (ECL) on film.

**Immunohistochemistry**

Mice were treated for 9 days with L-DOPA (10 mg/kg in combination with 7.5

mg/kg benserazide), anaesthetized and perfused 4 hrs post-injection on day 9.  Details can be found in the Materials and Methods section of the main manuscript.  Fixed tissue slices were incubated with anti-EGFP (Aves Lab, GFP-1020) and anti-Atf3 (Santa Cruz, sc-188) following the same protocol as described in the Materials and Methods section of the main text where the Image acquisition is also described.
